# Notes on Hospital Practice

**Published:** 1877-12-20

**Authors:** 


### NOTES ON HOSPITAL PRACTICE.

### ULCER OF THE STOMACH.

Nitrate of silver, in the form of pills, should be given in full doses half an hour before meals. If there be pain, opium, hydrocyanic acid, and chloroform may be administered. An exclusive milk diet is the best. All solid food must be avoided. At the time of hemorrhage, absolute rest must be insisted upon ; pieces of cracked ice should be swallowed. Monsel's solution, tannic or gallic acid, should be given internally ; morphia may be administered by the mouth, and ergotin hypodermically, and all food given by enemata for the time.

### LUMBAGO.

Manipulation must be applied to the lumbar region of the spine, so as to restore mobility. To subdue the painful condition of the muscle, injections of one-eightieth of a grain of atropia, and one-eighth of a grain of morphia, well diluted, should be made well into the body of the muscle. Great care must always be had in the administration of morphia and atropia to nursing women, as belladonna is the most powerful anti-galactagogue known, and too large doses of morphia not rarely affect the child through its milk. The local application of blisters, iodine and croton oil, together with the internal administration of iodide of potassium, often does good.

### INCIPIENT PHTHISIS.

Among the most important hygienic measures are good food, healthful outdoor exercise, which will expand the chest, and an equable climate, such as may be found in the south of California, New Mexico, or the southern and western slopes of Colorado. Sea voyages, such as a cruise to some tropical ocean, and not sailing about in an inclement climate, as many consider the term to mean, are most plainly beneficial. If these ways of regaining lost health be out of the question, and the patient be compelled to stay at home, inhalation of compressed air may be tried with success. Counter-irritation may be applied over the seat of disease, and cod-liver oil, the syrup of the iodide of iron, arsenic, and the hypoposphites of lime, soda and iron administered internally.

### IDIOPATHIC EPILEPSY.

As a general rule of treatment in epilepsy, all the existing causes of an attack, such as mental excitement, overeating, indigestible articles of diet, should be avoided. As concerns medicinal agents, the bromides are of especial value as controlling remedies. The initial dose should be twenty to sixty grains, thrice daily, the dose to be increased in size until either the paroxysms stop, or *bromism* is produced.

### TETANUS.

Systematic feeding of patients with liquid and strengthening food at short intervals has been employed with very good results. The food must be given at intervals of every two or three hours, and should consist mainly of milk, with a small quantity of alcohol. In severe cases all solid food must be avoided. As for medicines, the patient must be brought well under the influence of the bromide of potassium by an initial dose of two drachms to half an ounce, to be followed by half a drachm to a drachm every three or four hours. To force sleep at night, give at bedtime thirty grains of chloral, with some opium. Chloral also may be used, when necessary, in daytime. Nitrite of amyl and chloroform should not be used steadily, but may be employed from time to time to stop violent spasms. If *bromism* be produced, chloral and opium should be relied on, or cannabis indica may be substituted for the bromides. Where there is much cerebral congestion, a blister is put on the nape of the neck.

### TUBERCULAR LARYNGITIS.

Th[illegible] local application of pure nitric aci[illegible] or of strong solutions of nitrate of [illegible]er, is of great value. For the œdema astringent solutions, such as the sulphate of zinc, copper or alum may be recommended. Gargles and inhalations can be used for the cough. The following formula will be found of value:

R. Tinct. benzoini comp., fl. dr ij;

Glycerinæ, fl. oz. ss;

Aquæ q. s. ad fl. oz. iv. M.

Sig.—To be used as a gargle.

Inhalations of steam, vapor of hops, or conium, are sometimes successful as palliatives. Counter-irritation may be applied externally to the larynx in the shape of small blisters. To relieve the sense of fulness, lozenges of krameria, hæmatoxylon, or tannic acid are prescribed. In desperate cases, tracheotomy must be performed [see *Gleanings*, p. 23], and a metal tube worn, thus putting the much-irritated larynx at rest.

### CHRONIC INFLAMMATION OF BOWELS.

Individual symptoms must be borne in mind. Alcohol, green vegetables, fruits and meats, must be refrained from. Buttermilk, beef juice, milk with lime-water, and light farinaceous foods are the safest articles of diet. The clothing should be amply sufficient, and all excessive exposure avoided. Among medicines the proper mode of treatment is that by nitrate of silver in pill form, one-third of a grain from one to two hours after meals. The treatment must be long persevered in to effect a permanent cure.

### CATARRHAL JAUNDICE.

In the treatment of this complaint, early efforts must be directed to the allaying of the irritation of the mucous membrane of the stomach and duodenum. Do not begin by acting on the liver with cholagogues and mineral acids. All exposure must be avoided. A tumblerful of some one of the alkaline mineral waters should be taken twice daily, and nitrate of silver, with small doses of the extract of belladonna, given in pill form. Belladonna prevents spasmodic irritation of the ducts. Where there is much local irritation, blisters may be applied over the gall-duct and gall-bladder. After irritation has subsided, mercurials or mineral acids come into play.

### SACCHARINE DIABETES.

The diet must be modified by the exclusion of all sugary and starchy elements of food. The most valuable drug is opium, used in large doses for a long time. Ten grains may be given daily without producing the slightest drowsiness. Under this treatment the amount of urin passed daily will greatly diminish, and the proportion of sugar gradually grow less.

### CHRONIC ARTICULAR RHEUMATISM.

The most successful mode of treatment has been by manipulation of the anchylosed joints, and counter-irritation applied to the nerve-trunk higher up the leg. The continued current with the positive pole placed over the point of tenderness, and the negative pole higher up the nerve, may also be employed. When the foot is affected, a shoe should be constructed which shall take the strain off the painful joint, and throw the weight of the body on the outside of the foot (this for rheumatism of the joints of the foot, of course). Where there is a decided rheumatic diathesis, the persistent use of the following prescription is followed by advantageous effects:

R. Pulv. guaiaci resin., gr. x;
Potass. iodidi, gr. x;
Tinct. colchici semen, fl. dr. ss;
Aq. Cinnamomi,
Syrupi, aa q. s. ad fl. oz. i. M.

Sig.—A desertspoonful to a tablespoonful thrice daily.

ADENITIS

Is treated by continued injections of dilute ergotin in the substance of the inflamed gland.

ACUTE GASTRO-HEPATIC CATARRH.

In these cases, if a malarial nature be suspected, it is well to begin with full doses of quinia. If, however, the gastro-hepatic symptoms are prominent, the quinine treatment should be postponed for twenty-four hours, and from five to ten grains of blue mass given, followed by a saline. Then, when the liver and stomach have been well acted upon, quinia should be given by the rectum, so as to avoid gastric irritation. Four suppositories, of five grains each, should be given at intervals of from two to three hours. The diet should be restricted, and febrifuges and subacids given, and the skin sponged with cold water if the fever be severe.

TYPHOID FEVER.

Beginning with the second week of the disease, when the abdominal symptoms have fully set in, one-quarter of a grain of nitrate of silver, and from one-sixth to one-half of a grain of the watery extract of opium, with one-twelfth of a grain of belladonna, are exhibited in pill form, three times a day, after meals. Very little stimulus is used. Mild and beef tea are the only articles proper of food allowed. Quinia is given with other tonics. Fever is reduced by frequent spongings of the entire body with cool water. When the high fever resists sponging, cool baths are employed.—*Medical Times.*

